# Corrosion of the Welded Aluminium Alloy in 0.5 M NaCl Solution. Part 2: Coating Protection

**DOI:** 10.3390/ma11112177

**Published:** 2018-11-03

**Authors:** Andrey S. Gnedenkov, Sergey L. Sinebryukhov, Dmitry V. Mashtalyar, Igor E. Vyaliy, Vladimir S. Egorkin, Sergey V. Gnedenkov

**Affiliations:** 1 Institute of Chemistry of FEB RAS, 159 Pr. 100-letiya Vladivostoka, 690022 Vladivostok, Russia; sls@ich.dvo.ru (S.L.S.); madiva@inbox.ru (D.V.M.); igorvyal@gmail.com (I.E.V.); egorkin@ich.dvo.ru (V.S.E.); svg21@hotmail.com (S.V.G.); 2 School of Engineering, Far Eastern Federal University, 8 Sukhanova St., 690950 Vladivostok, Russia

**Keywords:** aluminium alloy, weld interface, plasma electrolytic oxidation, composite polymer-containing coating, SPTFE

## Abstract

The high electrochemical activity of the aircraft 1579 aluminium alloy with a welded joint and the necessity of the coating formation to protect this material against corrosion as well as to increase the stability of the weld interface in the corrosive medium has been previously established. In this work, two suggested methods of protective coating formation based on plasma electrolytic oxidation (PEO) in tartrate-fluoride electrolyte significantly increased the protective properties of the welded joint area of the 1579 Al alloy. The electrochemical properties of the formed surface layers have been investigated using SVET (scanning vibrating electrode technique) and SIET (scanning ion-selective electrode technique), EIS (electrochemical impedance spectroscopy), OCP (open circuit potential), and PDP (potentiodynamic polarization) in 0.5 M NaCl. The less expressed character of the local electrochemical processes on the welded 1579 Al alloy with the composite coating in comparison with the base PEO-layer has been established. Polymer-containing coatings obtained using superdispersed polytetrafluoroethylene (SPTFE) treatment are characterized by the best possible protective properties and prevent the material from corrosion destruction. Single SPTFE treatment enables one to increase PEO-layer protection by 5.5 times. The results of this study indicate that SVET and SIET are promising to characterize and to compare corrosion behaviour of coated and uncoated samples with a welded joint in chloride-containing media.

## 1. Introduction

In the first part of this work (Part 1) [1], the corrosion behaviour of the bare 1579 aluminium alloy sample with a welded joint was examined through scanning vibrating electrode technique (SVET), scanning ion-selective electrode technique (SIET) studies, immersion experiments, and corrosion products’ characterization. In summary, all the obtained results indicate the high corrosion activity of the Al alloy with a welded joint and the necessity of the coating formation to protect this material against intensive corrosion destruction and to increase its stability in the corrosive media.

In [1], we found that the presence of Mg as an alloying element in 1579 Al alloy sufficiently shifts the bulk pH values to the alkaline range as a result of the intensive dissolution of Mg. This effect decreases the stability of the 1579 Al alloy in the aggressive media. The area of the welded joint was found to be a zone of the corrosion process activation due to the presence of microdefects in the morphological structure.

This work (Part 2) deals with the formation and protective properties’ investigation of the coatings based on the plasma electrolytic oxidation (PEO) using localized electrochemical techniques (SVET and SIET) and global traditional electrochemical methods (electrochemical impedance spectroscopy with equivalent electrical circuit spectra fitting and potentiodynamic polarization).

Many alternative technologies for the corrosion protection of aluminium alloys are presently under study [2,3,4]. Jiang et al. formed thick epoxy coatings (~100 ± 10 μm) on AA2024 aluminium alloy [2,3]. Among the methods of protective coating formation on the surface of Al alloys, one of the most promising methods is plasma electrolytic oxidation [5,6,7,8,9,10,11,12,13,14,15]. Serdechnova et al. grew layered double hydroxides on PEO-pretreated AA2024 Al alloy [6]. Agureev et al. obtained PEO-layers on Al composites without additives and alloyed with copper in a silicate-alkaline electrolyte under an alternating current electrical mode [5]. Shoaei-Rad et al. used PEO to form ZrO_2_–Al_2_O_3_ composite layers with a pore size of 40–300 nm on the Al substrates [10]. Mohedano et al. developed voltage-controlled PEO-coatings and loaded the Zn–Al layered double hydroxides film with a corrosion inhibitor (vanadate), which was grown in situ on the coatings to provide active protection to the PEO layers on AA2024 aluminium alloy [13]. Egorkin et al. formed protective coatings on 5754 Al alloy with high mechanical and protective properties using PEO in a mixed electrolyte with microsecond polarizing current pulses [14]. Chen et al. showed that PEO can effectively inhibit dissimilar aluminium alloy welded parts against galvanic corrosion [15]. The properties of the formed surface layers depend on the polarization mode of PEO, electrolyte composition, and chemical composition and structure of the treated alloy [16,17,18,19,20,21]. Matykina et al. studied the influence of an anodizing pre-treatment in sulphuric acid on PEO of 99.99% aluminium in silicate electrolyte under a constant current [16]. Monfort et al. established that coatings produced by anodizing aluminium above the sparking voltage at 5 A dm^−2^ in potassium hydroxide/sodium metasilicate electrolytes consisted of an aluminium-rich inner part formed by growth of anodic alumina near the metal/coating interface and a silicon-rich outer part formed by deposition of silica gel on the coating surface [17]. Stojadinovic et al. used optical emission spectroscopy, atomic force microscopy, scanning electron microscopy with energy dispersive X-ray spectroscopy, and X-ray diffraction to characterize the morphology, composition, and microhardness of oxide coatings formed during direct current PEO of cold-rolled 99.999% purity aluminium samples in sodium tungstate [18].

There are numerous works dedicated to the modification of PEO-layers to improve their corrosion protection [22,23,24,25,26,27,28,29,30,31,32,33]. Zhang et al. used PEO as a pretreatment method to form calcium stearate based hydrophobic coatings [22]. Wen et al. used combined surface mechanical attrition treatment and the PEO process to obtain the ceramic coating on a modified nanocrystalline layer of 2024 Al alloy [23]. Ivanou et al. formed hybrid coatings by sealing PEO-layers with hybrid epoxy-silane formulation [24]. Lu et al. introduced SiO_2_ nanoparticles to PEO-coatings to seal the porosity and to provide a wider range of coating compositions [26]. Ghafaripoor et al. formed a composite coating on 7075 Al alloy via plasma electrolytic oxidation in silicate base electrolyte containing α-Al_2_O_3_ particles [31]. In [32], a duplex coating was obtained using PEO treatment of AA 6082 Al alloy, with subsequent formation of MoS_2_/Sb_2_O_3_/C chameleon solid lubricating coating as a top layer. A TiO_2_- and Al_2_O_3_-dense composite PEO film was generated on the substrate of Al 6061 alloy in a sodium silicate electrolyte containing dissolved TiO_2_ powder [33]. The promising method of PEO-layer improvement by means of formation of the composite polymer-containing coating using superdispersed polytetrafluoroethylene (SPTFE) was suggested for magnesium alloys [34]. It was shown that treatment of PEO-coatings by SPTFE enabled one to improve significantly both protective and antifriction properties of the magnesium alloy surface [34]. This method can also be applied to protect aluminium alloys against corrosion and mechanical damage. Application of SPTFE enables one to fill PEO-coating pores with the polymer and to form an additional compact barrier layer that will significantly decrease aggressive ions’ penetration to the substrate, reduce the metal ions’ release into the solution, and improve specimen stability in corrosion-active media [34].

In the present work, methods of the protective coating formation on the 1579 aluminium alloy (which is commonly used in aircraft application) surface with a welded joint are suggested. Such coatings can be characterized by enhanced protective properties. The electrochemical and protective properties of the formed surface layers have been thoroughly investigated.

The objective of the present work was to demonstrate, using SVET and SIET methods, the less expressed character of the local corrosion processes on the welded 1579 Al alloy with the polymer-containing coating in comparison with the base PEO-layer.

The novelty of this work is related to corrosion protection development of the weld interface, being a defect zone and stress concentrator. The method of composite layer formation using a combination of the PEO process with SPTFE treatment to protect the welded alloy against corrosion destruction was used in the current work for the first time.

## 2. Materials and Methods

### 2.1. Samples

In the present work, just like in [1], the 1579 Al alloy (Table 1) with a welded joint was used as a sample for study. The detailed process of sample preparation can be referred to [1].

The PEO-coating was formed on the surface of the aluminium alloy in the electrolyte of the following composition: C_4_H_4_O_6_K_2_∙0.5H_2_O, 20 g L^−1^, and NaF, 0.6 g L^−1^ in the unipolar galvanostatic PEO mode at *j* = 0.9 A cm^−2^. Potassium tartrate hemihydrate of the chemical formula, C_4_H_4_O_6_K_2_∙0.5H_2_O, was suggested earlier to form the coatings with high protective properties on aluminium alloys [35]. It was established [35] that potassium tartrate and sodium fluoride addition into the electrolyte increased its stability over time and resulted in formation of a uniform elastic film based on Al oxide and Al fluoride on the Al alloy substrate. The duty cycle was 100%. The polarization frequency was 300 Hz (pulse duration of 3.3 ms, no pauses). An H150-3000 Chiller Smart device (LabTech Inc., Hopkinton, MA, USA) was applied to control and maintain the temperature of the electrolyte (15 °C) during the oxidation process. An automated control system connected to a personal computer with appropriate software was used to control the electrical parameters. The PEO-process duration was equal to 150 s. A conventional reversible thyristor rectifier was used as a power supply [36]. The thickness of the obtained coating was about 10 µm.

To obtain the composite polymer-containing coating, the following treatment of the PEO-layer surface by means of Forum^®^ SPTFE was suggested to increase its protective properties and hydrophobicity and to decrease the coating porosity that enables one to reduce the corrosion intensity. The method of composite coating formation used in this experiment was described in detail elsewhere [34]. SPTFE was obtained in the process of fluoroplastic waste heat destruction by thermogradient synthesis in the Institute of Chemistry of the Far Eastern Branch of the Russian Academy of Sciences. 15 wt. % SPTFE powder suspension in the isopropanol was used to form the composite coatings. The polymer was applied to the base PEО-layer by the dip-coating method. The exposure time of the specimen to the suspension was up to 10–15 s. Then, the specimen was dried under ambient conditions for 20 min. The temperature and duration of thermal treatment of the specimen with composite coatings after the dip-coating process were 350 °C and 15 min, respectively. At the end of the process, the sample with polymer-containing coating after heat treatment was cooled under ambient conditions down to 25 °C. In the present work, a single polymer treatment of the specimen was used.

It is known that the microstructure of Al alloys and their mechanical properties could change at elevated temperatures. Airbus recommended the limit for the thermal post-treatment of AA2024 of 120 °C. In the present work, we studied the concept of polymer use to increase PEO-coatings’ protection. In the case of successful idea implementation, we will use another low-temperature fraction of SPTFE (with the melting point below 120 °C) or other polymers, like polyvinylidene fluoride (PVDF), and study the polymer treatment effect on the Al alloy’s mechanical properties.

### 2.2. Electrochemical Measurements

#### 2.2.1. SVET and SIET Measurements

In the present work, to investigate the evolution of the protective properties after PEO and SPTFE-treatment, the SVET/SIET system (Applicable Electronics, New Haven, CT, USA) was applied. Details of SVET and SIET measurements in this work are the same as in [1].

Quasi-simultaneous SVET/SIET measurements [37] were made on the aluminium alloy samples with protective coatings. The investigated area of the aluminium alloy with protective layers did not exceed 6 mm^2^ (after isolation with wax). The development of the corrosion process on the surface of the welded joint area of the 1579 aluminium alloy with coatings was monitored using SVET/SIET methods during specimen exposure to the corrosion-active media (98 h for PEO-coated specimen and 500 h for the polymer treated PEO-layer on Al alloy).

The SVET/SIET tests were repeated on two similar specimens to attain reproducibility and reliability of the obtained results.

The method of calculating total anodic and cathodic currents or the sum of these currents and making the plot of these values versus time [38,39] was used in this work and was described previously [1]. In this work, we present the plot of the sum of the total anodic and cathodic currents’ evolution versus time as well as the plots of the maximum and minimum pH as a function of time and ∆pH (the maximum pH difference between anodic and cathodic values) evolution with time for the PEO coated sample, the specimen with composite polymer-containing coating. The curves for the sample without coating were well described in [1], and are presented in this work only for comparison.

#### 2.2.2. Electrochemical Impedance Spectroscopy and Potentiodynamic Polarization Measurements

**Figure 1:**
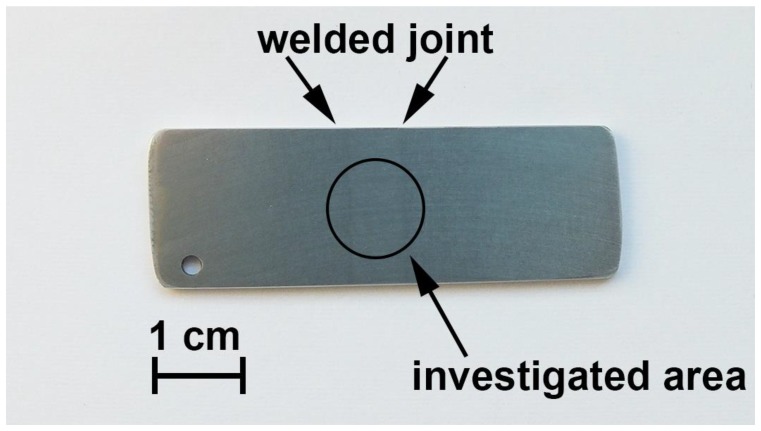
The photo of the 1579 aluminium alloy sample plate with a welded joint. The sample was made for electrochemical tests (open circuit potential (OCP), electrochemical impedance spectroscopy (EIS), potentiodynamic polarization (PDP)) (the investigated area is limited by circle).

Electrochemical properties of the specimens with and without protective layers were investigated using the potentiodynamic polarization (PDP) and electrochemical impedance spectroscopy (EIS) methods by means of the electrochemical system, 12558WB (Solartron Analytical, Hampshire, UK). This system consisted of an FRA 1255B frequency response analyzer and an SI 1287 electrochemical interface connected to the computer. Electrochemical tests were carried out in 0.5 M NaCl solution (pH = 7) in a Model K0235 Flat Cell three-electrode cell (Princeton Applied Research, Oak Ridge, TN, USA) at room temperature. The aluminium alloy specimens (plates) of a size of 20 mm × 50 mm × 1 mm for electrochemical measurements were used (Figure 1). In all these experiments, the exposed apparent area of the specimens to electrolyte was equal to 1 cm^2^. All the electrochemical parameters were calculated per this visible exposed area.

A platinum mesh was used as a counter electrode. The saturated calomel electrode (SCE) was used as a reference electrode. Before electrochemical measurements started, the specimens were immersed in 0.5 M NaCl solution for 120 min to reach the steady state and free corrosion potential, *E*_C_, fixing. The sweep rate of PDP measurements was equal to 0.167 mV/s. The specimens were polarized in the anodic direction from the potential of *E* = *E*_C_ − 250 mV up to *E* = *E*_C_ + 250 mV. The Levenberg-Marquardt (LEV) method was used to fit the measured PDP curve (i.e., current density, *I*, vs. potential, *E*) from *E*_C_ − 250 mV to *E*_C_ + 150 mV, according to the equation:

(1) $$I=I_{C}\left(10^{\left(E-E_{C}\right)/\beta_{a}}+10^{-\left(E-E_{C}\right)/\beta_{c}}\right),$$

which determined the best fit values of corrosion current density (*I*_C_), corrosion potential (*E*_C_), and anodic and cathodic slopes of the polarization curve (*β*_a_ and *β*_c_) [36,40,41]. To perform EIS measurements, a sinusoidal signal of the 10 mV (rms) amplitude was applied. The spectra were recorded at an open circuit potential (OCP) in the frequency ranging from 1 MHz down to 0.1 Hz with a logarithmic sweep of 10 points per decade. The OCP was recorded during 120 min of specimen immersion in the electrolyte to achieve the equilibrium, and then the last OCP value was held potentiostatically for EIS measurements. Thereby, the sample was in a steady state during EIS spectra recording. In another test, the OCP was also recorded to study the potential values changing during 24 h of the sample immersion in the electrolyte. The experiment control was realized by means of the CorrWare/Zplot software. The OCP, PDP, and EIS data processing was carried out using the CorrView/ZView software. The corrosion tests were repeated on three specimens for reproducibility and reliability. The measurement error did not exceed 5%.

### 2.3. Cross-Section Preparation

The cross-section of the studied Al alloy with a welded joint area for SVET/SIET measurements was prepared using methodology presented in [1]. After cross-sections were made, PEO and composite coatings were formed.

## 3. Results and Discussion

### 3.1. Study of the Localized Corrosion Process on the 1579 Al Alloy Sample with PEO-Coating

**Figure 2:**
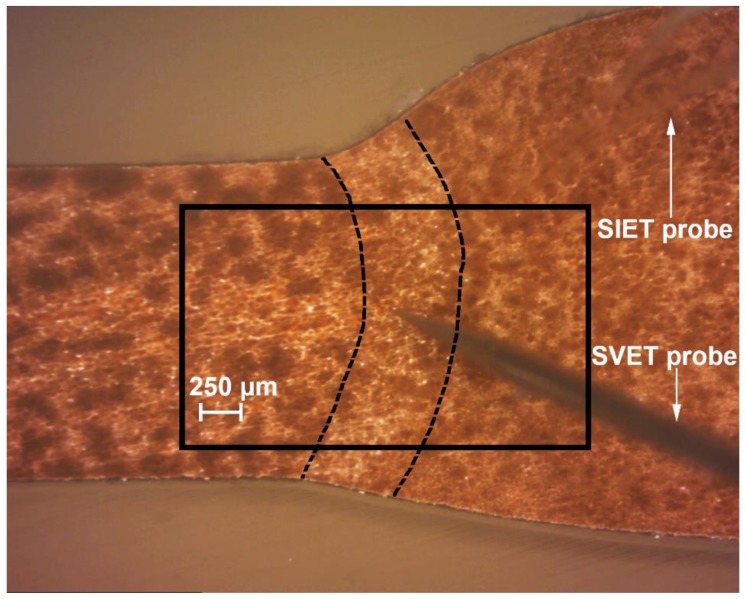
The optical image of the investigated welded joint area of the 1579 aluminium alloy sample with coating obtained using plasma electrolytic oxidation (PEO) before scanning vibrating electrode technique (SVET) and scanning ion-selective electrode technique (SIET) experiments. The investigated area is outlined by the frame and the border of the weld interface of sample is marked by a dotted line.

To protect the sample against corrosion destruction, the 1579 aluminium alloy specimen with a welded joint was processed by the PEO method. To observe the corrosion activity evolution in the area of the welded joint with PEO-coating in comparison with the sample without coating, the SVET and SIET experiments were performed. Figure 2 depicts the optical image of the investigated welded joint area. The investigated area is outlined by a frame and the weld interface of the sample is marked by dotted line (Figure 2).

**Figure 3:**
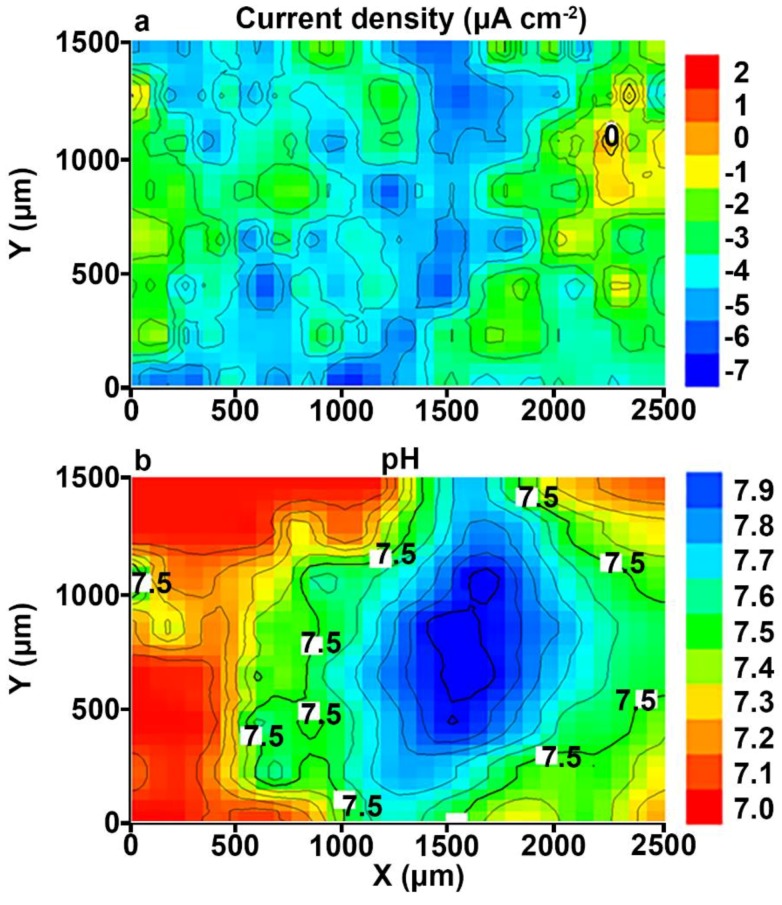
The (**a**) SVET and (**b**) SIET diagrams of the current density and pH distribution at the surface of the welded joint area of the 1579 aluminium alloy sample with PEO-coating after 510 min of exposure to 0.5 M NaCl. The weld interface is a cathodic zone.

The SVET and SIET can detect minor changes of the electrochemical activity (on a microscale level) on the surface of the investigated sample (Figure 3). The cathodic activity can be found on the weld interface itself and the neighbouring area of the welded joint (SVET map), but the intensive cathodic process was realized at the weld interface, which is confirmed by the SIET map (dark blue area). The dark blue cathodic area (zone with lower values of the current density and with higher values of the pH) at the weld interface can also be seen on the SVET map. A small discrepancy between the SVET and SIET maps can be related to low corrosion activity (low values of the current density) and, therefore, to low expression of the cathodic and anodic zones (anodic zone is an area with higher values of the current density and with lower values of the pH, red-orange area) as well as to some ion concentration mixing by probes due to the scanning process. In order to carry out the comparative analysis of the samples with PEO-coating (Figure 3) and without coating (Figures 3 and 4b in [1]), the SVET and SIET diagrams are presented after 510 min, since the corrosion activity of the PEO-treated sample did not significantly change during this period of time. The welded joint zone became a cathodic one after formation of the PEO-coating on the specimen surface (Figure 3).

Despite the uniform elements’ distribution in the aluminium alloy and welded joint area studied by the Energy Dispersive X-ray Analysis in [1], the results obtained by SVET and SIET measurements indicate to different occurrence of electrochemical processes on the surface of the material. The reason of the electrochemical activity of the weld interface is related to morphological peculiarities of this area and the presence of microdefects, which were detected by SVET/SIET measurements (Figures 3 and 8 in [1]). After PEO treatment, these defect zones were coated as a result of the PEO-layer formation, and the welded joint zone became a cathodic one (Figure 3). Due to the presence of the microdefects in the morphological structure of the welding joint area, the coating obtained by PEO on the surface of this area is denser and of higher quality than on the surface of the alloy. The effect of changing the polarity of the welded joint area from an anodic to cathodic one (value of the current density is down to −7 μA cm^−2^) is a result of the material protective properties’ increase after the PEO process. The other part of the sample plays an anodic role, with low electrochemical activity, because the values of the anodic current density did not exceed 2 μA cm^−2^ after 510 min of the specimen exposure (Figure 3a). The reason of low anodic current density uniformly distributed over the surface of the investigated material is related to unopened pores of the PEO-coating, which constitutes one of the peculiarities of such a type of coatings [34,36,42,43,44,45,46,47]. The maximum current density difference between anodic and cathodic values (∆*i_z_*, *z* stands for the vertical axis of the SVET probe vibration) for the Al sample without coating after 510 min of the specimen exposure to 0.5 M NaCl solution (Figures 3 and 4b in [1]) was equal to 180 μA cm^−2^. As compared to the sample with PEO-coating, this parameter did not exceed 9 μA cm^−2^ (Figure 3a). This result indicates high protective properties of the Al alloy with PEO-coating, especially in the welded joint area at the initial stage of the sample exposure. The SIET method also has registered a cathodic area at the welded joint zone (Figure 3b). The reactions, (4) and (6), described in [1], result in hydrogen evolution and local alkalization at the welded joint area. The pH values in cathodic and anodic zones for the sample with the PEO-coating are 7.9 and 7.0, respectively (Figure 3b), and 6.2 and 5.6 for the sample without coating (Figures 3 and 4b in [1]). This result indicates low rates of the reaction (3) in [1] for the PEO-coated sample, the electrolyte acidification process, and the whole corrosion process.

**Figure 4:**
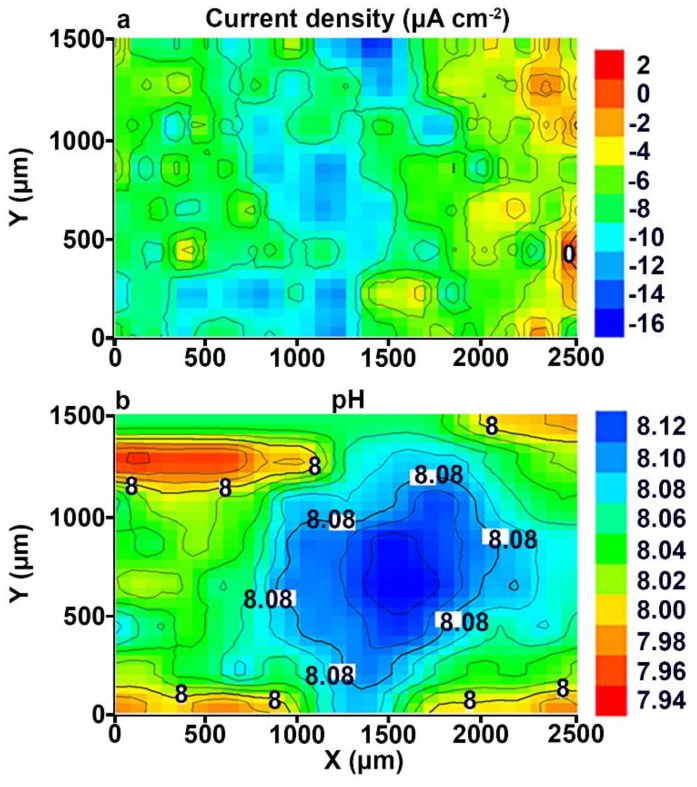
The (**a**) SVET and (**b**) SIET diagrams of the current density and pH distribution on the surface of the welded joint of the 1579 aluminium alloy sample with PEO-coating after 87 h of the exposure to 0.5 M NaCl. The welded joint area is a cathodic zone.

In order to compare the corrosion activity of the samples with (Figure 4) and without PEO-coating (Figure 8d in [1]) at the end of the experiment, the durations of the SVET and SIET experiments were limited to 87 h (the same as for the Al alloy sample without coating) of the specimen exposure to 0.5 M NaCl solution.

Since we know the time of high corrosion intensity for the Al alloy sample without coating (81–87 h) (Figure 8c,d in [1]), we used this experimental time for the PEO-coated sample to make a fair comparison of the corrosion properties changing. The electrochemical activity of the Al alloy sample with PEO-coating slightly changed during the 87 h. According to the SVET and SIET data (Figure 4), the welded joint area of the sample with coating, after 87 h of the exposure, is still a cathodic zone. The maximum value of the anodic current density did not change (2 μA cm^−2^), thus indicating the absence of the intensive corrosion process on the surface of the studied specimen. The values of current density in the cathodic zone increased from −7 μA cm^−2^ (Figure 3a) up to −16 μA cm^−2^ (Figure 4a). This is the result of the intensification of the cathodic reaction (4) in [1] and, therefore, the anodic area increase on the sample surface occurred (Figure 4a). The pH values in the cathodic and anodic zones increased up to 8.12 and 7.94 (Figure 4b), respectively. This effect is related to the increase of the pH level of the electrolyte, as a result of the cathodic reaction (4) in [1]. ∆*i_z_* for the uncoated Al alloy sample increased during the experiment time up to 400 μA cm^−2^ after 81 h of the sample exposure (Figure 8c in [1]) and decreased down to 120 μA cm^−2^ (due to corrosion product deposition) at the end of the experiment (87 h of sample exposure) (Figure 8d in [1]). ∆*i_z_* for the PEO-coated sample did not virtually change during the experiment and became 18 μA cm^−2^ (Figure 4a).

This result indicated a significant protection of the Al alloy sample against the corrosive chloride-containing media by means of the PEO method. The PEO-coating decreases the intensity of corrosion on the surface of the studied material. The area of the welded joint, which is an activator of the corrosion process in contact with the aggressive environment, became a cathodic zone, and, therefore, the corrosion destruction of the studied material was prevented during the experimental time.

**Figure 5:**
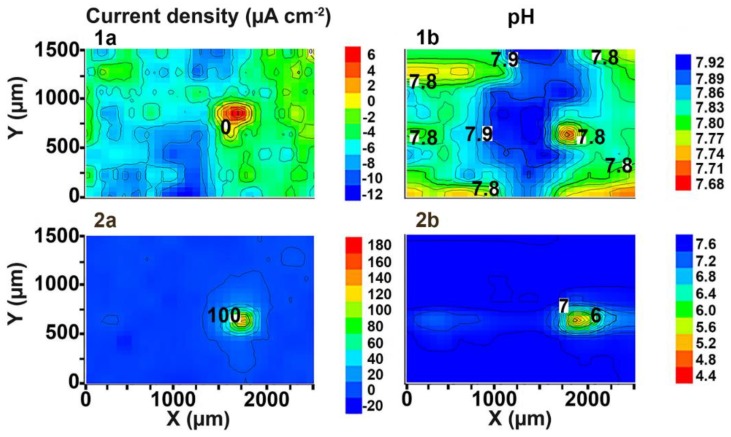
The (**a**) SVET and (**b**) SIET diagrams of the current density and pH distribution on the surface of the welded joint of the 1579 aluminium alloy sample with PEO-coating after (**1a**,**1b**) 90 and (**2a**,**2b**) 98 h of the exposure to 0.5 M NaCl. There is formation and development of the corrosion-active zone on the PEO-coating.

The experiment duration was increased to achieve the moment of PEO-coating degradation on the surface of the 1579 aluminium alloy specimen. The pitting formation was registered after 90 h of the sample exposure by means of SVET/SIET (Figure 5(1a,1b)).

**Figure 6:**
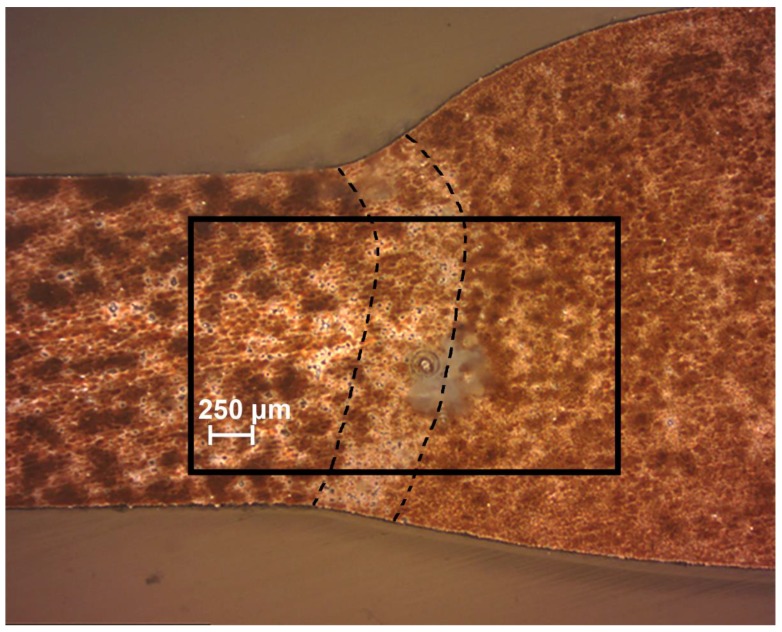
The optical image of the investigated welded joint area of the 1579 aluminium alloy sample with PEO-coating after SVET and SIET experiments (after 98 h of specimen exposure). The investigated area is outlined by a frame and the border of the weld interface of the sample is marked by a dotted line. There is a pitting at the weld interface.

The current density value in the anodic zone was equal to 6 μA cm^−2^ at the initial stage of defect evolution (Figure 5(1a)). The pH values were shifted to the more acidic range (Figure 5(1b)). Development of the corrosion-active zone was monitored during the next few hours of the sample exposure. The values of the current density in the anodic area became 180 μA cm^−2^ (98 h) (Figure 5(2a)). The pH values in the anodic zone decreased from 7.68 (90 h) down to 4.4 (98 h) (Figure 5(2b)). A small difference of the anodic zone location between the SVET and SIET data are related to some ion concentration mixing by probes due to the scanning process. ∆*i_z_* attained 200 μA cm^−2^ (18 μA cm^−2^ before pitting (Figure 4a)) and ∆pH became 3.2 (0.18 before pitting (Figure 4b)) after 98 h of the sample exposure to 0.5 NaCl (Figure 5(2a)), thus indicating high intensity of the material corrosion destruction and development of the corrosion process under the PEO-coating. Figure 6 depicts the optical image of the investigated welded joint area after 98 h of specimen exposure. The investigated area is outlined by a frame and the weld interface of the sample is marked by the dotted line (Figure 6). Analysis of the image obtained by optical microscopy (Figure 6) shows that pitting formation occurred at the weld interface. Despite PEO-treatment, which increases the protective properties of the 1579 aluminium alloy, the weld interface, being a zone undergoing an intensive corrosion destruction due to the presence of microdefects in the morphological structure, is still the weakest place in such material. This result grounds the necessity of the PEO-coating modification process.

### 3.2. Study of the Localized Corrosion Process on the 1579 Al Alloy Sample with the Composite Polymer-Containing Coating

**Figure 7:**
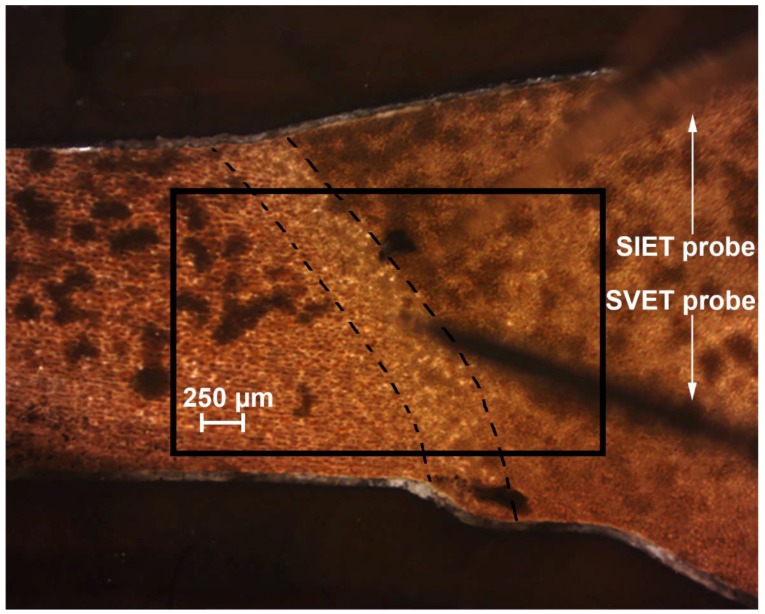
The optical image of the investigated welded joint area of the 1579 aluminium alloy sample with composite polymer-containing coating after SVET and SIET experiments (after 500 h of specimen exposure). The investigated area is outlined by a frame and the border of the weld interface of the sample is marked by a dotted line.

To obtain the layer with the best protective properties and to decrease the activity of the weld interface, the PEO-layer was treated by SPTFE. After the composite polymer-containing coating was formed, SVET and SIET were used to study changes in the corrosion activity of the welded joint with such a surface layer in comparison with the sample with the PEO-coating. The specimen was immersed in 0.5 M NaCl during 500 h. Figure 7 depicts the optical image of the investigated welded joint area of the sample with composite coating. The investigated area is outlined by a frame and the weld interface of the sample is marked by a dotted line (Figure 7).

**Figure 8:**
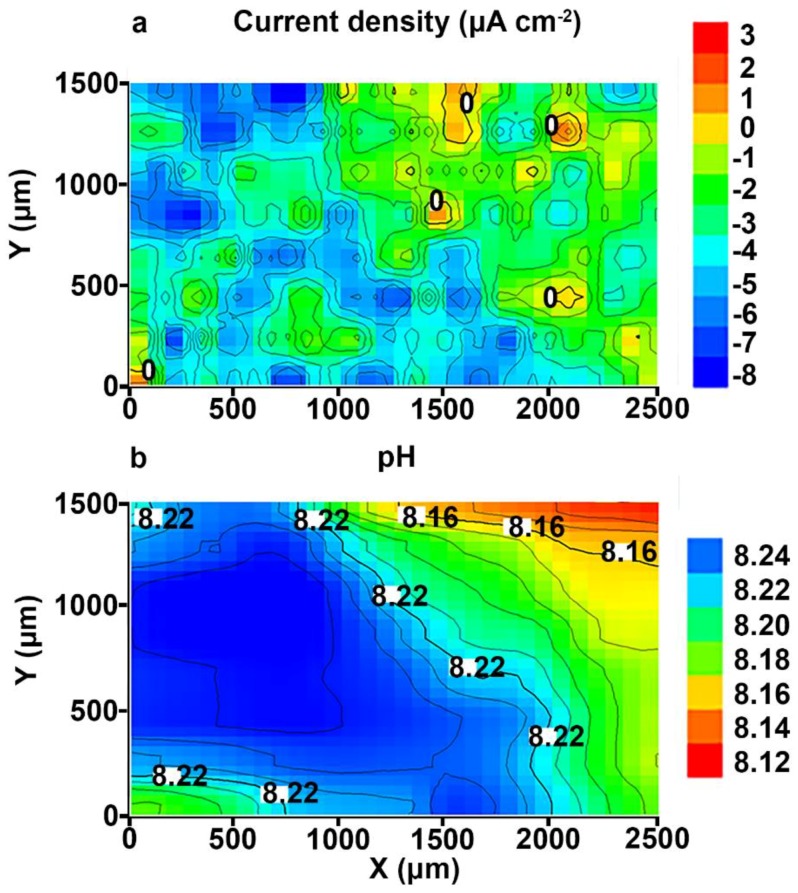
The (**a**) SVET and (**b**) SIET diagrams of the current density and pH distribution on the surface of the welded joint of the 1579 aluminium alloy sample with composite polymer-containing coating after 500 h of exposure to 0.5 M NaCl. The welded joint area is a cathodic zone.

During the experiment, the SVET and SIET could detect low electrochemical activity on a microscale level on the surface of the investigated sample. According to the SVET/SIET data, there were no pitting formation, coating degradation, and other significant electrochemical changes during the experiment. In order to demonstrate a substantial improvement of the protective properties of the aluminium alloy with a composite layer in comparison with (Figure 4) and without the PEO-coating (Figures 3 and 8 in [1]), the SVET and SIET diagrams after 500 h are presented (Figure 8), as the corrosion activity of the polymer-treated sample did not significantly change during this period. The welded joint zone is a cathodic one (Figure 8), just like after formation of the PEO-coating on the specimen surface (Figure 3 and Figure 4). ∆*i_z_* and ∆pH were about 11 μA cm^−2^ and 0.12 (Figure 8), respectively, which is even lower in comparison with the PEO-coating before pitting (18 μA cm^−2^ and 0.18) (Figure 4a). Low anodic and cathodic activities (3 μA cm^−2^ and −8 μA cm^−2^), as well as more alkaline values of the pH (from 8.12 up to 8.24) (Figure 8), are related to low rates of the reactions, (3)–(8), as described in [1], thus indicating the high protective properties of the composite coating. The cathodic zone at the weld interface (Figure 8) was still present after composite coating formation, which is related to partial pores filling and pore size decreasing through single polymer treatment of the PEO-coating surface. Therefore, such cathodic and anodic activities studied by the SVET/SIET (Figure 8) are the result of NaCl solution penetration through some non-sealed coating pores to the poreless PEO-layer during the sample exposure. To decrease such low values of the electrochemical activity, the multiplicity of SPTFE treatment should be increased to seal all PEO-coating pores. Nevertheless, even single SPTFE treatment enables one to significantly improve PEO-coating and increase protective properties (more than 5.5-fold) of the PEO treated Al alloy, which was corroborated by 500 h (90 h for PEO-coating) of sample exposure to 0.5 M NaCl solution based on the SVET/SIET data.

### 3.3. Corrosion Performance of the 1579 Al Alloy Samples

**Figure 9:**
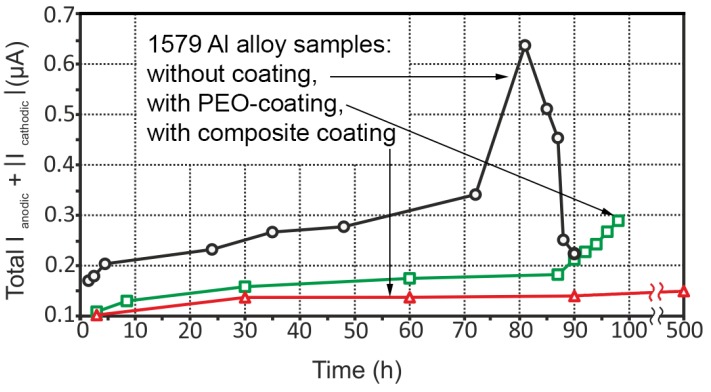
The evolution of the sum of the total cathodic and anodic currents for three aluminium alloy samples (without coating, with PEO-layer, with composite polymer-containing coating). The sample with the composite coating has the lowest values of the activity with the maximum value of total currents of 0.15 µA after 500 h of sample exposure.

The evolution of the sum of the total cathodic and anodic currents for three aluminium alloy samples (without coating, with PEO-layer, with composite polymer-containing coating) is shown in Figure 9. Descriptions of the corrosion behaviour for the sample without coating is presented in [1].

Similar tendencies are observed for each specimen: An increasing of the corrosion activity was registered during the exposure of the specimens. An almost twofold activity decrease was registered for the sample with the PEO-layer (the middle curve) during all the exposure time. After 87 h, total currents began to increase from 0.18 µA up to 0.29 µA due to the pitting formation process, which was discussed above (Figure 5). The sample with the composite polymer-containing coating (the lower curve) has the lowest corrosion activity with the maximum value of total current of 0.15 µA after 500 h of sample exposure.

**Figure 10:**
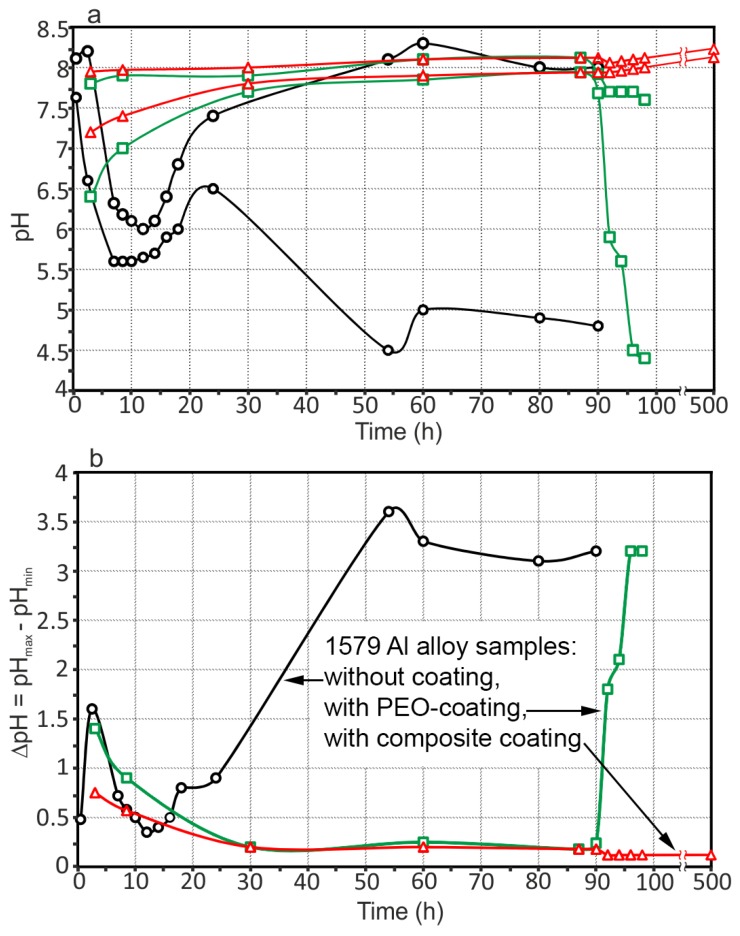
(**a**) Maximum and minimum pH as a function of time and (**b**) ∆pH evolution with time for 1579 Al alloy samples without coating, with a PEO-layer, and with composite polymer-containing coating. The sample with composite coating demonstrates the smallest variations of ∆pH, which is around 0.12–0.15 during all the exposure time.

To compare the evolution of the pH distribution for the three Al alloy specimens, the maximum and minimum pH values as well as the maximum pH difference between the anodic and cathodic values as a function of time are presented in Figure 10.

For the sample with the PEO-layer (square symbols), the maximum and minimum pH values increased (up to 7.9 and 7.0, respectively) within 30 h. The pH values were stable until 90 h, and then pitting formation took place (pH values decreased down to 4.4) (Figure 10). The sample with composite coating (triangle symbols) has alkaline values of the maximum and minimum pH, which slightly increased with time (8.24 and 8.12, respectively) (Figure 10a). The stability indication of the measured pH for each specimen is presented by ∆pH. Analyzing the pH difference between the maximum and the minimum pH at each time (shown in Figure 10b), the sample without coating varies the most, from 0.35 up to 3.60. ∆pH for the PEO treated sample is lower than that for the sample without coating: ∆pH = 0.24 before the pitting and 3.2 after pitting formation. The sample with composite coating demonstrates the smallest variations of ∆pH, which is around 0.12–0.15 during all the exposure time.

### 3.4. EIS, OCP, and PDP Studies of Electrochemical Properties of the 1579 Al Alloy Samples

**Figure 11:**
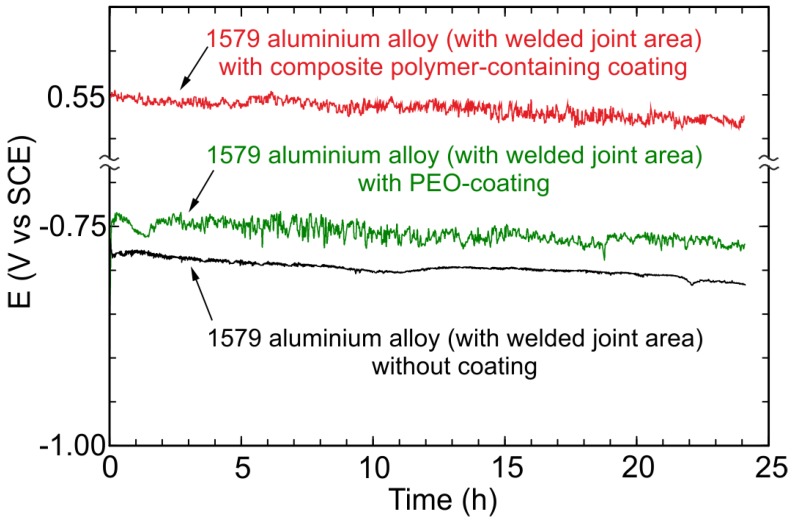
Open circuit potential changing versus time (24 h) for the 1579 aluminium alloy (with welded joint area) samples without coating (the lower curve), with PEO-coating (the middle curve), and with composite polymer-containing coating (the higher curve) obtained in 0.5 M NaCl solution.

To measure the electrochemical parameters of the 1579 aluminium alloy with and without protective coatings, the OCP, EIS, and PDP methods were used. The OCP evolution versus time (24 h) for the aluminium alloy specimens (including the welded joint zone) without coating (the lower curve), with the PEO-layer (the middle curve), and with the composite polymer-containing coating (the higher curve) obtained in 0.5 M NaCl solution are shown in Figure 11. Polymer treatment of the PEO-layer increases the potential values for the sample without coating and for the specimen with the PEO-layer, which indicates the substantial improvement of the coating protective properties. OCP changing during 24 h shows the stable electrode potential values, which is a result of the coating stability to the corrosion processes.

**Figure 12:**
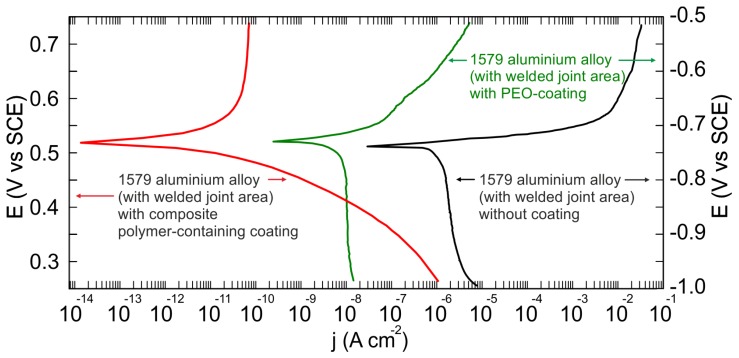
Polarization curves for the samples made of 1579 aluminium alloy (with welded joint area) without coating (the right one), with the PEO-coating (the middle one), and with the composite polymer-containing coating (the left one) obtained in 0.5 M NaCl solution.

Figure 12 depicts polarization curves for the aluminium alloy (with welded joint area) specimens without coating, with the PEO-layer, and with the polymer-containing coating. As calculated from the experimental data, the corrosion current density value for the polymer-containing coating was equal to 28 pA cm^−2^, which is more than four orders of magnitude lower than for the specimen without coating (1.4 µA/cm^2^) and more than two orders of magnitude lower than for the PEO-layer (10 nA/cm^2^) (Table 2).

The experimental data presented in Table 2 confirmed the SVET/SIET results, indicating protective properties’ improvement for the sample with the composite polymer-containing layer as compared to the uncoated and PEO treated samples. The sample with the composite polymer-containing layer has a lower corrosion current density and nobler corrosion potential values in comparison with the uncoated specimen and the sample with the PEO-layer (Table 2) (Figure 12). Cathodic and anodic Tafel slopes, *β*_c_ and *β*_a_, calculated from the polarization curve by LEV fitting [36,40,41] are also presented in Table 2. The value of the goodness of fit (*χ*^2^) did not exceed 0.1, which is a result of the nonideal specimen surface.

**Figure 13:**
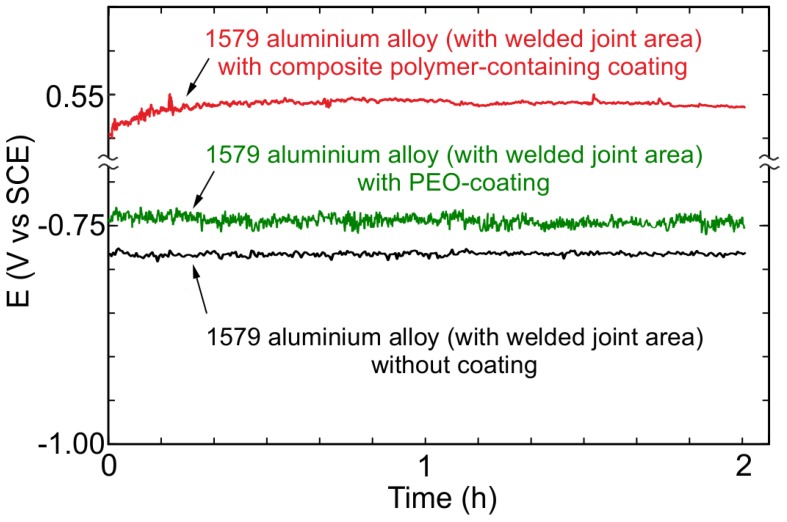
Open circuit potential changing versus time (2 h) for the 1579 aluminium alloy (with welded joint area) samples without coating (the lower curve), with PEO-coating (the middle curve), and with composite polymer-containing coating (the higher curve) obtained in 0.5 M NaCl solution. The last OCP value was held potentiostatically for EIS measurements.

**Figure 14:**
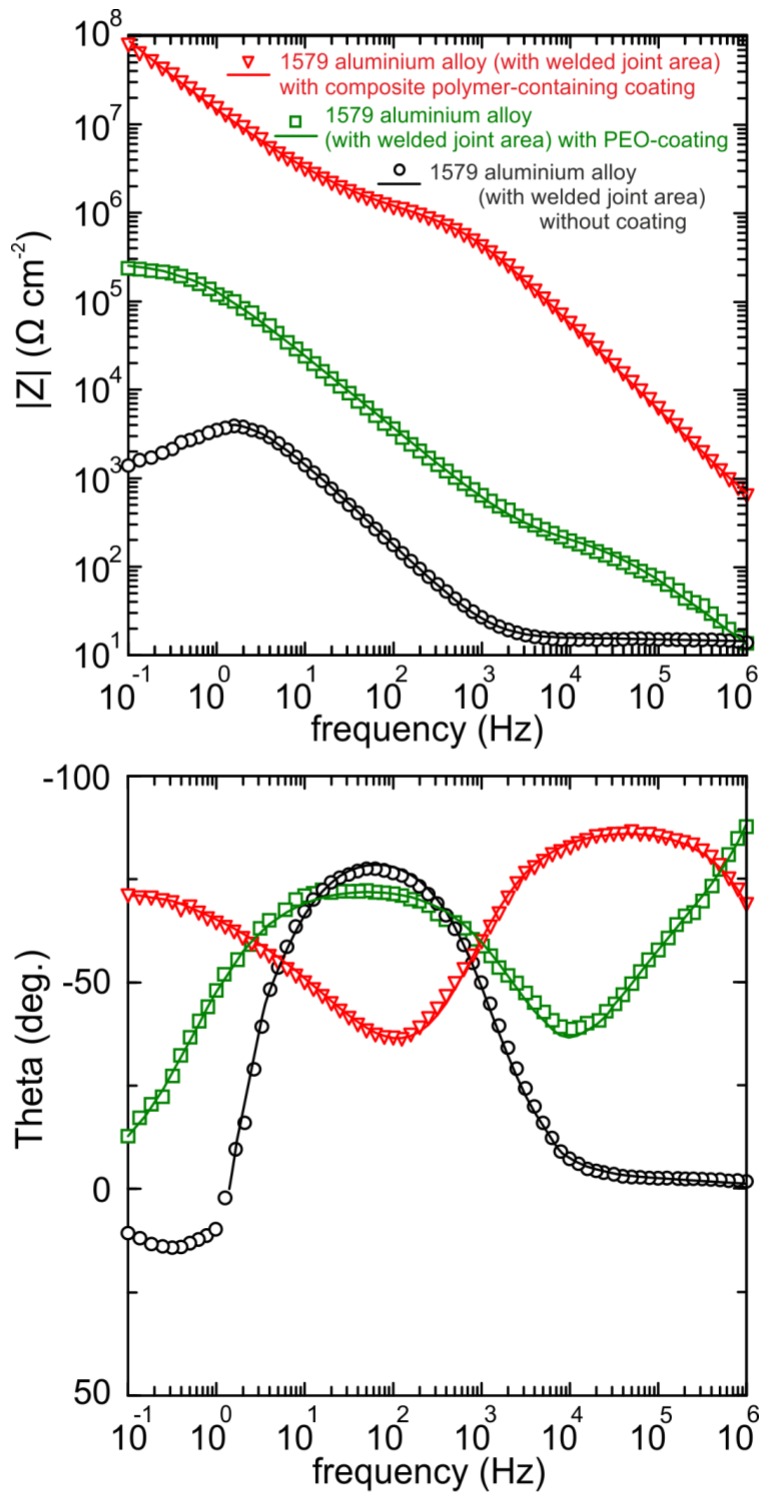
Impedance spectra for the samples made of 1579 aluminium alloy (with the welded joint area) without coating (circle symbols), with PEO-coating (square symbols), and with composite polymer-containing coating (triangle symbols) obtained in 0.5 NaCl solution. The graphs of the dependence of the impedance modulus (|*Z*|) and phase angle (*Theta*) on frequency (Bode plots).

**Figure 15:**
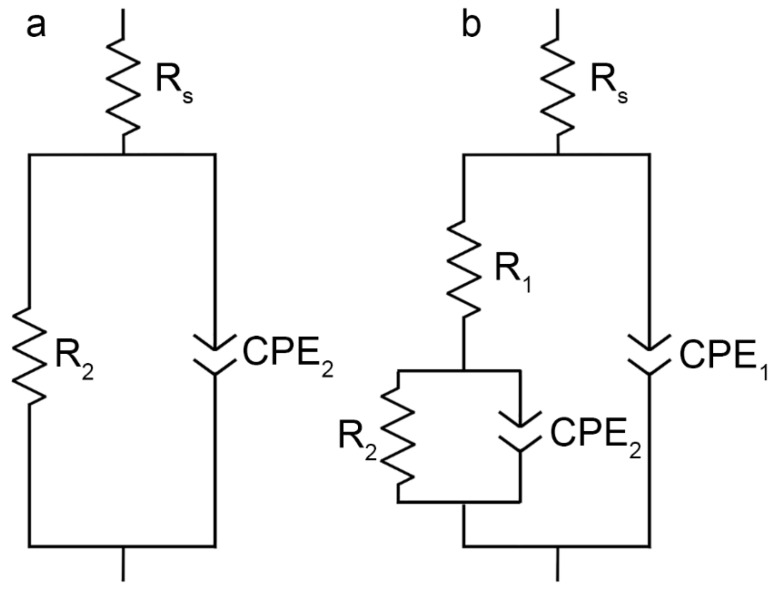
Equivalent electrical circuits, consisting of: (**a**) One *R-CPE*-circuit, and (**b**) two *R-CPE*-circuits. EEC were used for the EIS experimental results’ simulation (Figure 14) using fitting parameters (Table 3).

Before starting the EIS experiment, to achieve equilibrium, the OCP was recorded during 120 min of specimen exposure to the electrolyte (Figure 13), and then the last OCP value was held potentiostatically for EIS measurements. Therefore, the specimen was in a steady state during EIS spectra recording. OCP data obtained during 24 h (Figure 11) and 120 min (Figure 13) are in good agreement with each other. The EIS technique was used to study the specimen corrosion properties’ change after PEO treatment and after composite coating formation. The impedance spectra for Al alloy (1579 with the welded joint) with and without PEO-coating, and with composite polymer-containing coating presented in Figure 14 contain experimental results (scatter plot) and theoretical fitting curves (solid line), which simulate the experimental data, using equivalent electrical circuits (EEC) (Figure 15). The fitting results of the EIS experimental data (Figure 14) are shown in Table 3.

Analysis of the impedance modulus’ dependence on frequency (Figure 14) confirms the results made on the basis of the PDP test. The impedance modulus value at low frequency (|*Z*|_f=0.1Hz_) for the specimen with composite polymer-containing coating is equal to about 93 MΩ cm^2^, which is more than two orders of magnitude higher than for the base PEO-layer (240 kΩ cm^2^) and by four orders of magnitude higher than for the uncoated sample (1.3 kΩ cm^2^) (Table 2). The values of impedance modulus for the sample without coating decreased at the low-frequency range from 1 Hz down to 0.1 Hz. This effect is related to the electrochemical reaction of dissolution of the uncoated aluminium alloy with partial destruction of its surface layers. The values of impedance modulus for the specimen with the composite layer and PEO-coated sample increased over the whole frequency range, thus indicating a higher corrosion stability and higher protective properties of the PEO-based layers, as compared to the bare Al alloy.

The phase angle (*Theta*) dependences on the frequency (Figure 14) reveal the morphological properties’ changes as well as specimen heterogeneity after the PEO-layer and polymer-containing coating formation. The EIS spectrum of the sample without coating has one time constant due to the presence of the natural oxide film on the surface, i.e., it can be described by EEC with one *R*_2_–*CPE*_2_-circuit (*CPE*—Constant Phase Element), where *R*_2_ is the charge transfer resistance and *CPE*_2_ is the double layer capacitance at the electrode/electrolyte interface (Figure 15a) [34]. The positive phase angle values at low frequencies are related to intermediate products’ adsorption as a result of the pitting corrosion process [48]. Due to surface layer corrosion destruction, the electrochemical behaviour at low frequencies was not reproducible. Thereby, the frequency range for analysis and experimental data fitting was intentionally limited, which is a common practice in EIS measurements [48]. The EECs suggested in this work are correlated to ones used by various research groups dealing with EIS data fitting [4,23,49,50,51].

There are two time constants for the spectrum of the specimen with the PEO-layer. The first time constant (from 10 kHz up to 1 MHz) with the phase angle maximum of −90° is responsible for the oxide layer geometric capacitance. The second one (from 1 Hz up to 10 kHz), which describes the poreless sublayer, has a phase angle maximum at −75°. To fit the impedance spectra of the specimens with the PEO-layer and composite polymer-containing layer with a high accuracy (*χ*^2^ = 1 × 10^−4^), the EEC with a serial-parallel connection of two *R*–*CPE*-chains was used (Figure 15b). In Figure 15b, the *R*_1_–*CPE*_1_ and *R*_2_*–CPE*_2_ circuits present the porous coating part and inner poreless sublayers, respectively. The spectrum for the sample with the polymer-containing layer has a second time constant, with the maximum at −75° in the lower frequency range (0.1 Hz), as compared to the base PEO-coating. This is a result of impedance magnitude growth for the composite polymer-containing layer [36,41]. The reason of CPE element use in the EEC data was described in detail elsewhere [36]. The presence of the second time constant for the specimen with the PEO-layer and composite coating indicates the more protective properties of these layers in comparison with the uncoated sample.

The data obtained by EEC fitting of the EIS experimental results (Table 3) are corroborated by the PDP measurements (Table 2). Geometric and poreless sublayer thicknesses of the composite layer were increased in comparison with the PEO-coating. This is a result of coating pores’ sealing by SPTFE. It is corroborated by the parameter, *Q* (*CPE*_1_ and *CPE*_2_), decreasing (Table 3) for the polymer-containing coating. As a result of polymer treatment of the base PEO-coating, the pore channel inlets, as well as the quantities of surface microdefects, were decreased. The value of the parameter, *R*_1_, which simulates the resistance of electrolyte in pores, increased, thus confirming the aforementioned process of pore narrowing for the composite layer. The fact of SPTFE penetration into the PEO-coating pores was established and proven elsewhere [34]. The heterogeneity level of surface layers on Al alloy samples was presented by the parameter (*n*) value in Table 3. The closer the value (*n*) is to 1, the more homogeneous is the surface. The values of the poreless layer resistance (*R*_2_) also increased along with the sample surface type changing from the specimen without coating and PEO-layer to the composite coating (Table 3). This fact indicates the reinforcement of the dense poreless sublayer after polymer-containing coating formation.

The calculated EEC parameters indicate the better resistance of the composite coating to the corrosion process as compared to the base PEO-layer. According to the fitting results of the EIS data by EEC (Table 3), the total resistance of the composite polymer containing layer, (*R*_1_ + *R*_2_) = 681 MΩ cm^2^, is more than three orders of magnitude higher than that for the base PEO-layer (270 kΩ cm^2^). The latter also indicates higher protective properties of the polymer-containing layer.

The latter result corroborated a significant increase of protective properties of the Al alloy sample with a welded joint against corrosion in chloride-containing media through PEO-layer and composite polymer-containing coating formation. The PEO-coating on the surface of the aluminium alloy prevents the material from intensive corrosion destruction during the experimental time. To make the Al alloy more stable in the corrosive environment and to decrease the electrochemical activity of the weld interface, the coating obtained by the PEO-method should be additionally modified through SPTFE polymer treatment. Polymer-containing coatings provide aluminium alloy with reliable corrosion protection.

To sum up, the SVET and SIET findings described in the present paper show that a composite protective coating reduces the corrosion activity of treated material in chloride-containing media. These results also indicate that single SPTFE treatment is sufficient to significantly improve the PEO-layer protective properties. Here, the results obtained by means of localized methods enabled us to choose the appropriate mode and multiplicity of PEO-coating polymer treatment. Presented in Part 1 [1] and Part 2, SVET and SIET, in combination with conventional OCP, EIS, and PDP methods, provided the information about local (steps, kinetics, and mechanism of the corrosion processes at a microscale level) and global electrochemical behaviour of the Al alloy without coating, and with the base PEO-layer and composite polymer-containing PEO-coating.

## 4. Conclusions

Methods of the protective coating formation on the aircraft 1579 aluminium alloy surface were suggested to decrease the electrochemical activity of the weld interface.

Analysis of the corrosion behaviour and protective properties of coated 1579 aluminium alloy specimens with a welded joint revealed the following conclusions:

1. The coating obtained by the PEO method in tartrate-fluoride electrolyte decreases the corrosion intensity of the studied sample. The current density difference between anodic and cathodic areas for the PEO treated sample decreased by more than one order of magnitude as compared to the bare 1579 aluminium alloy, from 400 μA cm^−2^ down to 18 μA cm^−2^, according to SVET and SIET.
2. According to SVET/SIET data, the area of the welded joint, which is an activator of the corrosion process in contact with the aggressive environment, became a cathodic zone due to the PEO-layer, which prevented the corrosion destruction of the studied material.
3. The promising method of PEO-layer modification using superdispersed polytetrafluoroethylene (SPTFE) was applied. It has been established that single SPTFE treatment enables one to significantly improve the protective properties of the PEO-coated 1579 Al alloy (more than 5.5-fold), see the SVET/SIET data.
4. Electrochemical methods (OCP, EIS, and PDP) showed a high level of sample protection using PEO-based coatings. The value of the impedance modulus at low frequency as well as that of the corrosion current density corroborated a significant corrosion inhibition in chloride-containing media for the Al alloy sample with a welded joint through the PEO-layer (240 kΩ cm^2^, 10 nA/cm^2^) and composite polymer-containing coating (93 MΩ cm^2^, 28 pA/cm^2^) formation.
5. The combined results of Part 1 and Part 2 of this study indicate that SVET and SIET represent useful methods to characterize and to compare corrosion behaviour of the coated and uncoated samples with a welded joint in chloride-containing media. The data obtained using localized and conventional electrochemical methods enabled us to choose the appropriate multiplicity of PEO-coating treatment with polymer.

## Figures and Tables

**Table 1:** Chemical composition of tested 1579 Al alloy in weight (%).

Mg	Zn	Cu	Si	Fe	Zr	Sc	Cr	Ni	Ti	Al
6.78	0.62	0.14	0.51	0.15	0.13	0.13	0.17	0.10	0.02	balance

**Table 2:** Main electrochemical performances of the 1579 aluminium alloy (include welded joint area) samples with different treatment. Data were obtained by means of the potentiodynamic polarization and electrochemical impedance spectroscopy methods.

Sample #	Type of Sample Surface	|*Z*|_f=0.1Hz_ (Ω cm^2^)	*I*_C_ (A/cm^2^)	*E*_C_ (V vs. SCE)	*β*_a_ (mV/Decade)	−*β*_c_ (mV/Decade)
1	Without coating	1.3 × 10^3^	1.4 × 10^−6^	−0.739	16.7	1114.8
2	PEO-layer	2.4 × 10^5^	1.0 × 10^−8^	−0.728	63.8	1320.0
3	Composite polymer-containing coating	9.3 × 10^7^	2.8 × 10^−11^	0.523	285.24	89.9

**Table 3:** Calculated parameters of the element of the equivalent electrical circuit for the samples made of the 1579 aluminium alloy (including the welded joint area) with different treatment. These parameters were used for a plot of fitting curves (solid lines in Figure 14).

Sample #	Type of Sample Surface	*CPE*_1_		*R*_1_ (Ω cm^2^)	*CPE*_2_		*R*_2_ (Ω cm^2^)
		*Q* (S cm^−2^ s^n^)	*n*		*Q* (S cm^−2^ s^n^)	*n*	
1	Without coating	–	–	–	1.2 × 10^−5^	0.95	4.3 × 10^3^
2	PEO-layer	4.4 × 10^−7^	0.76	2.7 × 10^2^	8.9 × 10^−7^	0.85	2.7 × 10^5^
3	Composite polymer-containing coating	3.7 × 10^−10^	0.97	7.9 × 10^5^	1.9 × 10^−8^	0.87	6.8 × 10^8^
